# Supplementary material for: Developmental Transcriptomic Analysis of the Cave-Dwelling Crustacean, Asellus aquaticus
Source: Genes (Basel). 2019 Dec 29;11(1):42. doi: 10.3390/genes11010042 (PMC7016750; doi:10.3390/genes11010042)
Supplement: Supplementary file 1 [file genes-11-00042-s001.zip › Supplemental Information.docx]

**Supplemental Figure Legend and Information**

**Supplementary Figure 1:** BUSCO analysis of *Asellus aquaticus* transcriptomes.  A BUSCO analysis was performed on all four transcriptomes prior to being filtered for transcripts greater than 1000bp in sequence.

Statistics for the four unfiltered transcriptomes

stats for Surface_Transcripts.fas
sum = 120457683, n = 113432, ave = 1061.94, largest = 9042
N50 = 1278, n = 31475
N60 = 1105, n = 41619
N70 = 948, n = 53385
N80 = 792, n = 67253
N90 = 606, n = 84468
N100 = 156, n = 113432
N_count = 1649
Gaps = 1287

stats for Cave_Transcripts.fas
sum = 127928737, n = 119569, ave = 1069.92, largest = 9717
N50 = 1305, n = 32308
N60 = 1123, n = 42874
N70 = 957, n = 55210
N80 = 790, n = 69892
N90 = 596, n = 88339
N100 = 158, n = 119569
N_count = 1615
Gaps = 1254

stats for Hybrid_Transcripts.fas
sum = 137138598, n = 143962, ave = 952.60, largest = 7928
N50 = 1107, n = 42508
N60 = 968, n = 55767
N70 = 839, n = 70979
N80 = 711, n = 88689
N90 = 558, n = 110278
N100 = 154, n = 143962
N_count = 2002
Gaps = 1558

stats for Integrated_Transcripts.fas
sum = 282052316, n = 293474, ave = 961.08, largest = 10318
N50 = 1150, n = 83477
N60 = 1000, n = 109794
N70 = 862, n = 140169
N80 = 718, n = 175929
N90 = 548, n = 220392
N100 = 154, n = 293474
N_count = 4587
Gaps = 3632
