# Supplementary figures and images for: Developmental Transcriptomic Analysis of the Cave-Dwelling Crustacean, Asellus aquaticus
Source: Genes (Basel). 2019 Dec 29;11(1):42. doi: 10.3390/genes11010042 (PMC7016750; doi:10.3390/genes11010042)

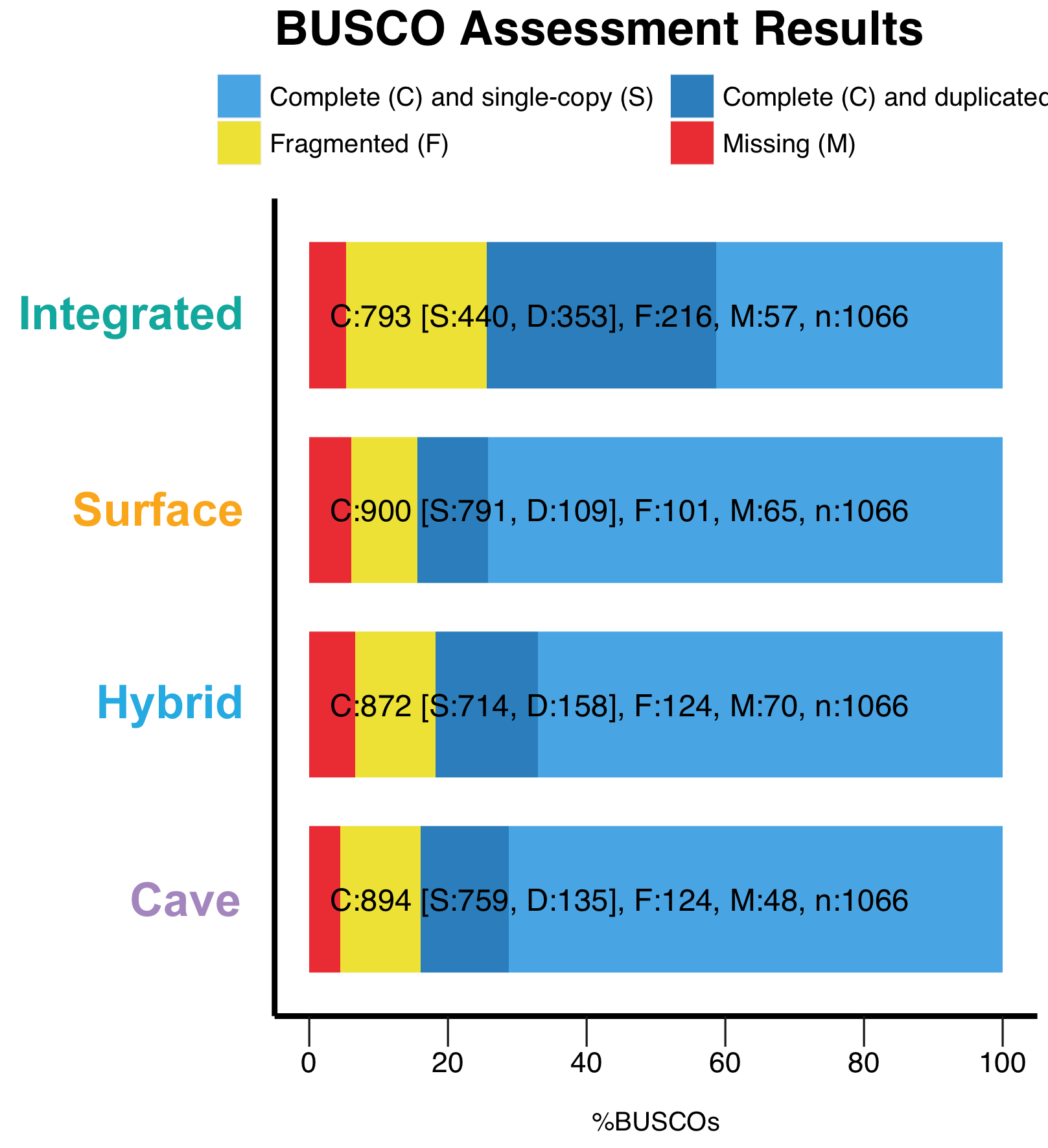

Supplement: Supplementary file 1 [file genes-11-00042-s001.zip › Supplementary Figure 1.tif]
